# RACIAL/ETHNIC DISADVANTAGE IN PARENTAL DEATH ACROSS THE LIFE COURSE, SOCIAL ISOLATION, AND HEALTH IN LATER LIFE

**DOI:** 10.1093/geroni/igac059.1367

**Published:** 2022-12-20

**Authors:** Rachel Donnelly, Zhiyong Lin, Debra Umberson

**Affiliations:** Vanderbilt University, Nashville, Tennessee, United States; University of Texas at San Antonio, San Antonio, Texas, United States; The University of Texas at Austin, Austin, Texas, United States

## Abstract

Prior research has not considered how exposure to parental death across the life course may contribute to lasting social isolation and, in turn, poor health among aging adults. The present study uses longitudinal data from the Health and Retirement Study (HRS; 1998-2016) to consider linkages of parental death, social isolation, and health (self-rated health, functional limitations) for Black, Hispanic, and White older adults. Findings suggest that exposure to parental death before later life is associated with higher levels of isolation, greater odds of fair/poor self-rated health, and more functional limitations in later life. Moreover, social isolation partially explains associations between parental bereavement and later-life health. Racial disparities in bereavement are central to disadvantage: Black and Hispanic adults are more likely to experience parent death early in the life course and this differential exposure to bereavement has implications for racial and ethnic disparities in social isolation and health throughout life.

